# Does Place of Residence or Time of Year Affect the Risk of Stroke Hospitalization and Death? A Descriptive Spatial and Temporal Epidemiologic Study

**DOI:** 10.1371/journal.pone.0145224

**Published:** 2016-01-22

**Authors:** Shamarial Roberson, Matthew Dutton, Megan Macdonald, Agricola Odoi

**Affiliations:** 1 Florida Department of Health, Bureau of Chronic Disease Prevention, Tallahassee, Florida, United States of America; 2 Florida Agricultural and Mechanical University, Tallahassee, Florida, United States of America; 3 University of Tennessee, Knoxville, Tennessee, United States of America; Geisel School of Medicine at Dartmouth College, UNITED STATES

## Abstract

**Background:**

Identifying geographic areas with significantly high risks of stroke is important for informing public health prevention and control efforts. The objective of this study was to investigate geographic and temporal patterns of stroke hospitalization and mortality risks so as to identify areas and seasons with significantly high burden of the disease in Florida. The information obtained will be useful for resource allocation for disease prevention and control.

**Methods:**

Stroke hospitalization and mortality data from 1992 to 2012 were obtained from the Florida Agency for Health Care Administration. Age-adjusted stroke hospitalization and mortality risks for time periods 1992–94, 1995–97, 1998–2000, 2001–03, 2004–06, 2007–09 and 2010–12 were computed at the county spatial scale. Global Moran’s I statistics were computed for each of the time periods to test for evidence of global spatial clustering. Local Moran indicators of spatial association (LISA) were also computed to identify local areas with significantly high risks.

**Results:**

There were approximately 1.5 million stroke hospitalizations and over 196,000 stroke deaths during the study period. Based on global Moran’s I tests, there was evidence of significant (p<0.05) global spatial clustering of stroke mortality risks but no evidence (p>0.05) of significant global clustering of stroke hospitalization risks. However, LISA showed evidence of local spatial clusters of both hospitalization and mortality risks with significantly high risks being observed in the north while the south had significantly low risks of stroke deaths. There were decreasing temporal trends and seasonal patterns of both hospitalization and mortality risks with peaks in the winter.

**Conclusions:**

Although stroke hospitalization and mortality risks have declined in the past two decades, disparities continue to exist across Florida and it is evident from the results of this study that north Florida may, in fact, be part of the stroke belt despite not being in any of the traditional stroke belt states. These findings are useful for guiding public health efforts to reduce/eliminate inequities in stroke outcomes and inform policy decisions. There is need to continually identify populations with significantly high risks of stroke to better guide the targeting of limited resources to the highest risk populations.

## Introduction

Stroke occurs when there is loss of blood supply to a section of the brain [1]. More than 15 million people worldwide suffer from stroke with approximately one-third (5.5 million) being fatal [2]. Stroke is the second leading cause of death worldwide and is a significant public health concern in the United States. It is estimated that a stroke event occurs in the United States every 40 seconds, and someone dies from the condition every four minutes [3, 4]. Stroke places a considerable burden on the healthcare system in terms morbidity/mortality and associated financial/economic costs in addition to the fact that the disease drastically reduces life expectancy and quality of life [5]. The economic burdens of the disease in Florida and United States are estimated at $5.5 and $73.7 billion annually, respectively [6, 7]. Moreover, since the risk of stroke increases with age, it places a considerable burden on Florida’s healthcare system due to the state’s aging population.

Geographical disparities in stroke mortality risk have existed since the early 1940s [7]. Investigating these disparities and identifying geographic areas and population groups with significantly high risks of stroke is important for guiding public health prevention and control efforts. Globally, there are geographical differences in stroke burden with increasing risk being reported in Europe, the Americas, Asia as well as Africa [1]. There are reports of drastic geographical differences in case fatality rates and hospitalizations from stroke even within the European countries [8]. Studies have also reported that the prevalence of stroke in Latin America, India, and China is higher in urban areas than rural areas [9]. In the US, higher risks of the disease have been observed in the southeastern states, also called the “stroke belt”, compared to the rest of the country [10]. In the stroke belt, stroke mortality rates are approximately 20% higher than the rest of the United States.

Most previous efforts to understand geographical differences in stroke risk in many health departments, including Florida, have mainly mapped age-adjusted stroke hospitalization and mortality risks/rates. Very few studies conducted by public health departments to guide evidence-based control efforts to date have used spatial statistical approaches to investigate for evidence of statistically significant geographical clusters of stroke hospitalization and mortality risks. Moreover, the aging demographic of the US, and especially Florida, implies increased risk of chronic diseases such as stroke. Thus, such investigations would be the first step in assessing the burden and guiding the targeting of resources for disease prevention and control to populations most in need so as to reduce health disparities and improve health of the entire population. It is worth noting that identification of spatial clusters of stroke hospitalization and mortality risks is important for guiding public health prevention efforts, shaping policy and developing interventions to eliminate disparities [8]. Therefore studies are needed to investigate spatial patterns and clustering of stroke hospitalization and mortality risks so as to identify high risk areas and populations. The identification of areas with significantly high risk clusters will aid health officials in resource allocation and provision of health services so as to reduce stroke disparities [11]. Thus, the objective of this study was to investigate geographic and temporal disparities in stroke, and identify areas with statistically significant higher risk of stroke hospitalizations and deaths in Florida.

## Methods

### Study area and data sources

The study area encompassed the entire state of Florida which had a population of approximately 19 million in 2012. Over 17.9% of the population were over 65 years of age, a segment of the population known to have a higher risk of chronic diseases, including stroke. Although nearly 70% of Florida land is designated as rural, only approximately 9% of the population lives in rural areas. This segment of the population may not have timely access to emergency healthcare that stroke patients typically need. The overall racial/ethnic composition of Florida is approximately 80% white, 15% black, and 20% are of Hispanic origin.

Population estimates, used as denominators for calculation of mortality and hospitalization risks, were obtained from the Florida Legislature Office of Economic and Demographic Research. Mortality and hospital data covering the time period from 1992 to 2012 were obtained from the Florida Department of Health and the Florida Agency for Health Care Administration. Stroke deaths were identified by ICD-10 codes I60-I69 while hospitalizations were identified by ICD-9-CM codes 430–438. ArcGIS 10.1 software was used to link mortality and hospitalization data to county cartographic boundary files downloaded from the US census bureau [12].

### Data analysis

All descriptive statistical analyses were performed using SAS 9.4 [13]. Age-adjusted stroke hospitalization and mortality risks were calculated at the county level spatial scale using annual population as denominators and the US population as the standard population for direct standardization. All computed hospitalization and mortality risks were expressed as the number of cases per 100,000 persons. To assess changes in geographic disparities over time, the age-adjusted risks were computed for 7 different time periods: 1992–94, 1995–97, 1998–00, 2001–03, 2004–06, 2007–09 and 2010–12. Temporal patterns were assessed by plotting monthly hospitalization and mortality risks using Microsoft Excel 2010 [14].

#### Detection of spatial clusters

Global Moran’s I, implemented in GeoDa [15], was used to detect significant spatial autocorrelation of stroke hospitalization and mortality risks using a queen spatial weight. Additionally, Moran local indicators of spatial association (LISA) were computed to identify the locations of significant hotspots/clusters of stroke hospitalization and mortality risks also using queen spatial weights. Nine hundred ninety-nine Monte Carlo replications were used to assess statistical significance. When the simulated p-value was less than 0.05, the null hypothesis of no clusters was rejected.

#### Cartographic displays

All Cartographic manipulations and displays were performed in ArcGIS 10.1 [16]. Jenk’s optimization classification scheme was used to determine the critical intervals for mapping age-adjusted stroke hospitalization and mortality risks in choropleth maps. The choropleth maps were generated in three-year time periods: 1992–94, 1995–97, 1998–00, 2001–03, 2004–06, 2007–09 and 2010–12. Evidence of local hotspots/clusters of high hospitalization and mortality risks were displayed in ArcGIS 10.1 [16] using local Moran significance maps that identified local clusters of significantly high risks as well as those with significantly low risks.

## Results

### Spatial patterns of stroke hospitalization and mortality risks

**Fig 1:**
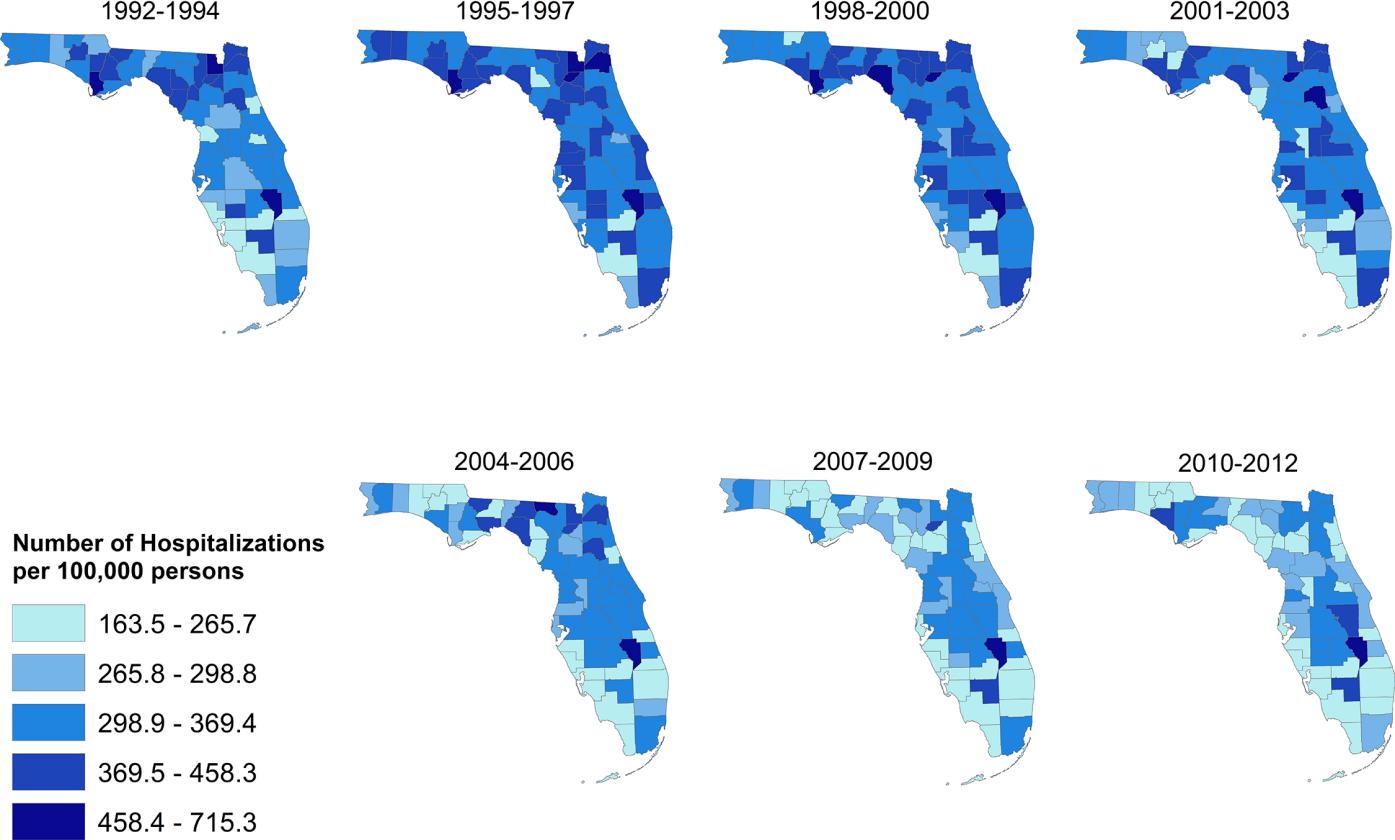
Spatial distribution of stroke hospitalization risks in Florida, 1992–2012.

The total number of stroke hospitalizations and deaths over the study period was 1,463,524 and 196,618, respectively. Age-adjusted stroke hospitalization risks varied by geographical region ranging from 163.5 to 715.3 per 100,000 persons and were consistently high throughout the state from 1992 to 2003 with a slight decrease from 2004 to 2012 (Fig 1). Higher hospitalizations risks tended to occur in the central and northern regions of the state while the lowest risks were observed in the south.

**Fig 2:**
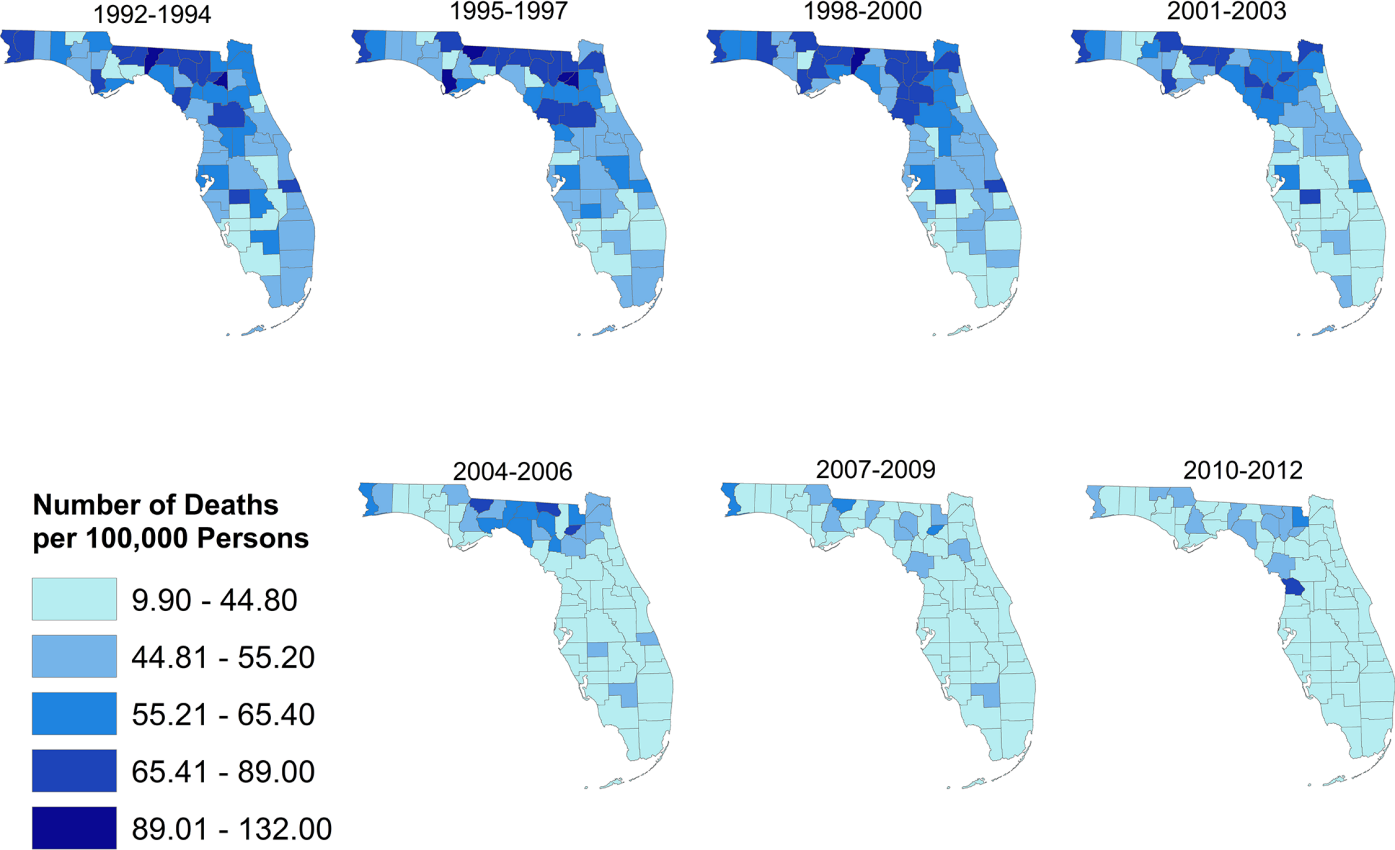
Spatial distribution of risk of death from stroke in Florida, 1992–2012.

Stroke mortality risks, on the other hand, ranged from 9.9 to 132 per 100,000 persons with the highest risks being observed in the north (Fig 2). The risks also tended to decrease over the study period as evidenced by the lighter shaded areas for time periods 2004–2006, 2007–2009 and 2010–2012.

### Geographic clusters of stroke hospitalization and mortality risks

#### Global evidence of clustering

Significant clusters of high hospitalization risks were identified only during the time period 1992–94 (Moran’s I = 0.212; p = 0.005). However, there were significant clusters of high mortality risks for all time periods investigated: 1992–94 (Moran’s I = 0.132; p = 0.003), 1995–97 (Moran’s I = 0.169; p = 0.001), 1998–2000 (Moran’s I = 0.240; p = 0.001), 2001–03 (Moran’s I = 0.204; p = 0.001), 2004–06 (Moran’s I = 0.267; p = 0.001), 2007–09 (Moran’s I = 0.111; p = 0.006), and 2010–12 (Moran’s I = 0.263; p = 0.001).

#### Local clusters

**Fig 3:**
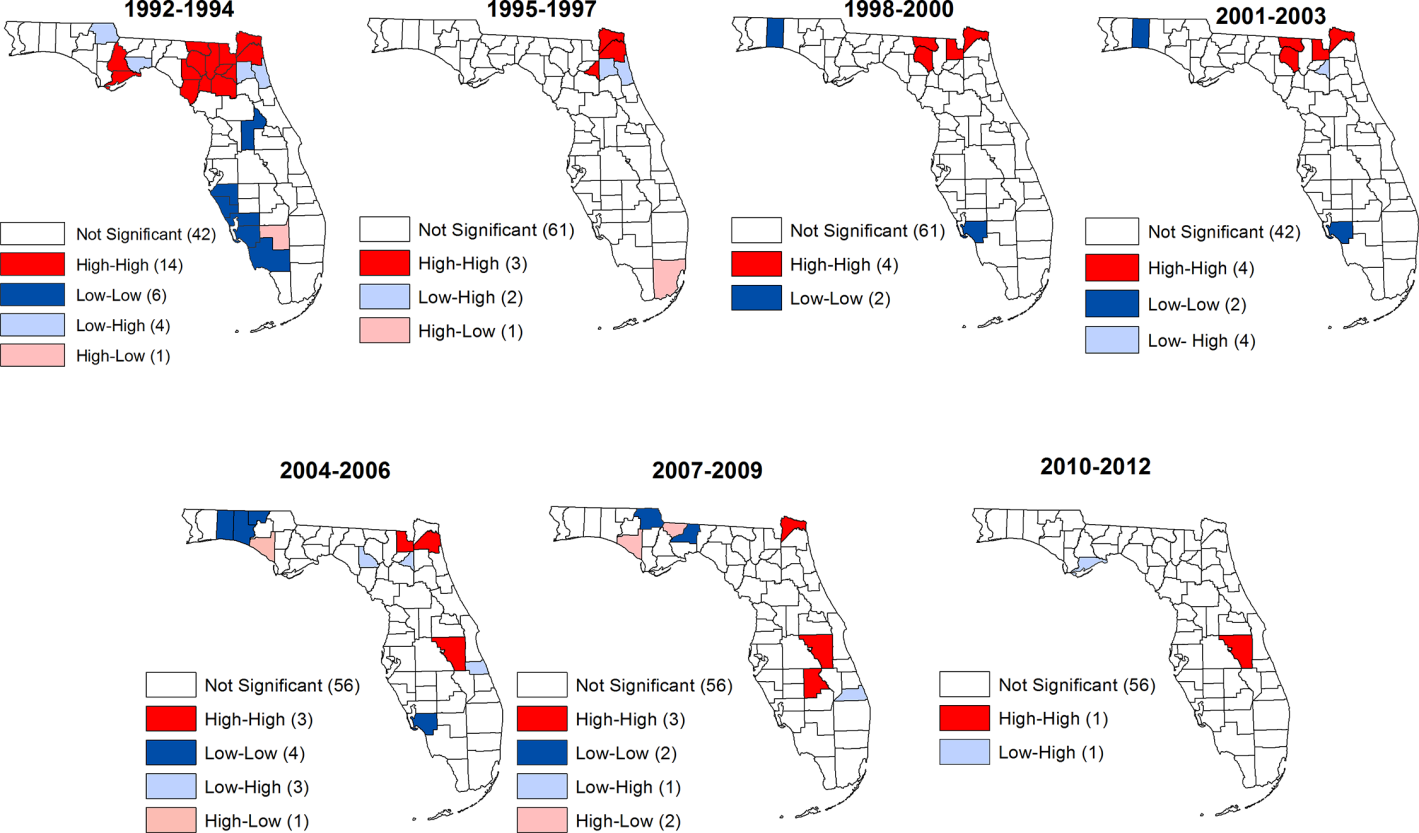
Spatial distribution of counties with significantly high or low stroke hospitalization risks in Florida, 1992–2012.

**Fig 4:**
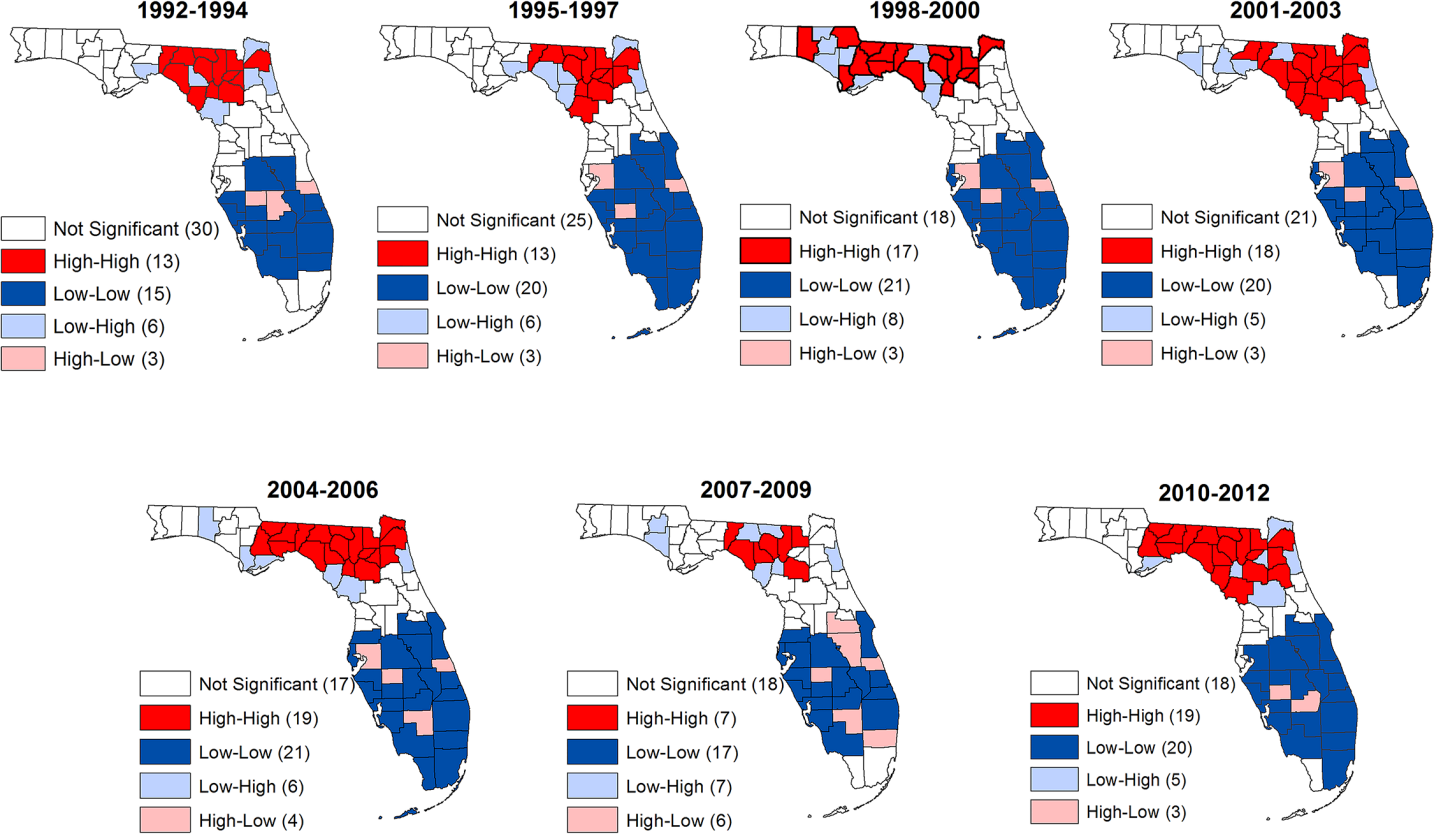
Spatial distribution of counties with significantly high or low risks of stroke deaths in Florida, 1992–2012.

Local indicators of spatial association (LISA) significance maps for stroke hospitalization and mortality risks revealed the four types of spatial associations (high-high, low-low, high-low, low-high) (Figs 3 and 4). These spatial associations constitute two forms of spatial autocorrelation: positive and negative spatial autocorrelations.

Positive spatial autocorrelations refer to associations between similar values (either high-high or low-low). Counties marked as high-high are those with high risks of either hospitalization or mortality that are surrounded by other counties with high risks (hospitalization or mortality). Similarly, counties marked as low-low are those with low risks of hospitalization/mortality risks and are surrounded by other counties with low hospitalization/mortality risks. Thus, these high-high and low-low counties exhibit positive spatial autocorrelation or clustering of either high risks (high-high) or low risks (low-low). There are also two forms of negative spatial autocorrelations which represent associations between dissimilar values: high-low and low-high. High-low counties are those with high hospitalization/mortality risks in areas surrounded by low values of the weighted average risks of the neighboring areas, whereas low-high signify low hospitalization/mortality risks in a county surrounded by high values of the weighted average risk of the neighboring counties. Significant negative spatial association is shown in blue while significant positive association is colored red. Areas with non-significant LISA values are blank (no color shading).

Significant high risk clusters of varying sizes were observed consistently from 1992 to 2009 in counties in the northeastern part of the state (Fig 3). Additionally, a significant high risk cluster was observed consistently in one county in the middle part of the state from 2001 to 2012. Notably, over the 21 years covered in this study, stroke hospitalization risks steadily decreased. Moreover, disparities also significantly decreased as evidenced by the fact that several clusters (both high and low risk clusters) were identified in 1992–1994 but only two clusters (one high risk cluster and the other low risk clusters) were identified in 2010–2012.

Similar to the results of hospitalization risks, significantly high mortality risks spatial clusters (high-high) were observed in the north of the study area (Fig 4). However, low mortality risk spatial clusters were observed in the middle and southern counties of the state. This pattern was consistent for all seven time periods investigated (Fig 4). Thus, although there seemed to be a decrease in disparities associated with hospitalization risks, disparities associated with mortality risks did not show evidence of change with the northern part of the state consistently having counties that formed part of the high mortality risk clusters while the central and southern counties consistently formed the low risk clusters.

### Temporal patterns

**Fig 5:**
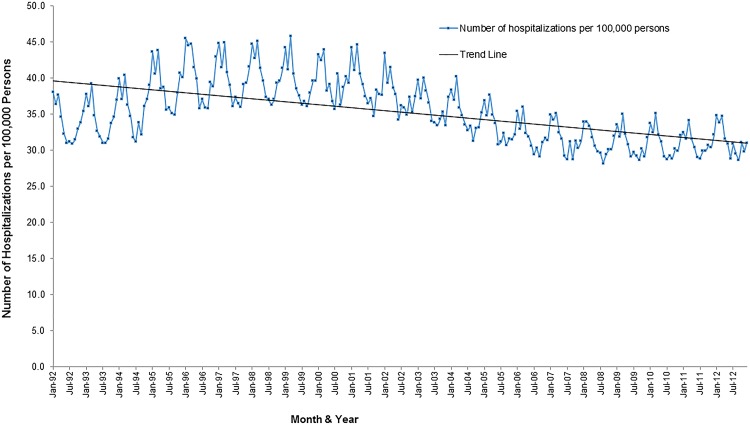
Temporal patterns of stroke hospitalization risks in Florida, 1992–2012.

Stroke hospitalization risks increased from 1992 to 2000 and decreased thereafter but remained relatively stable from about 2007 to the end of the study period (Fig 5). Seasonal patterns of stroke hospitalization risks were also observed with high risks being observed consistently in the cold winter months of January to March (Fig 5).

**Fig 6:**
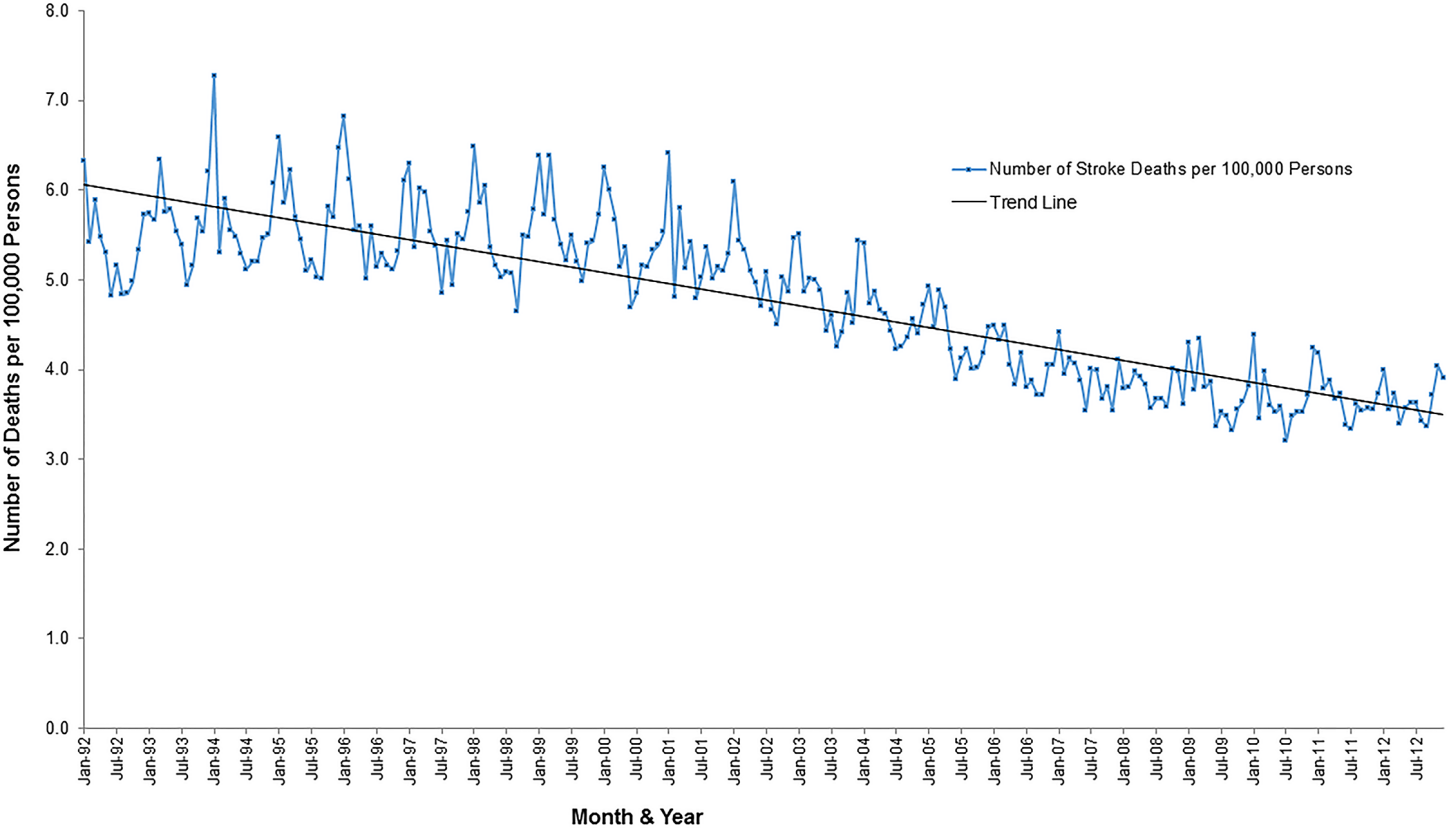
Temporal patterns in the risk of stroke deaths in Florida, 1992–2012.

Additionally, there were overall decreasing secular temporal trends for both hospitalization and mortality risks over the 21 year period (Figs 5 and 6). The highest stroke mortality risk was observed in the winter of 1995 (18.7 cases per 100,000 persons) and the lowest in the summer of 2010 (10.21 cases per 100,000 persons).

## Discussion

The north of the state, which had higher risks of both stroke hospitalization and mortality risks, is generally more rural than the south and therefore the results seem to suggest higher risks in the more rural areas compared to the more urban areas of the state. These results suggest that north Florida may need to be considered as part of the national stroke belt. Since many national campaigns on stroke interventions focus on stroke belt states, this region would definitely benefit from such programs. Since it is obvious that it is an area of need and that should be targeted for intervention. The findings of high risks in the north are similar to those from a study by Tassone et al [17] which reported higher stroke death rates in rural areas of Southeastern US. Another study performed in middle Tennessee by Odoi and Busingye also reported that rural census tracts tended to have significantly higher stroke mortality risks compared to urban census tracts. On the contrary, Pedigo Aldrich and Odoi [18], in a study performed in East Tennessee, did not find a significant association between stroke mortality risks at the neighborhood level and rural/urban status of the neighborhood [18]. Moreover, findings of studies from outside the United States have reported even more contrasting findings. For instance, a study conducted among older people living in Latin America, India and China revealed that the prevalence of self-reported stroke was much lower in rural areas of India, China and Peru than in urban areas of those countries [9]. Obviously no direct comparison can and should be made between our study and the above study since ours investigated mortality risk while the other investigated prevalence. However, mention of it is worthwhile in this discussion due to differences in the direction of the relationships. A separate study performed in India reported stroke prevalence to be lower (84–262 cases per 100,000 persons) in rural areas than in urban areas (119–145 cases per 100,000) with observed wide regional variations in case fatality rates [19]. That Indian study also noted that stroke centers in India are predominantly located in urban areas [19].

In the US, it has been reported that rural areas generally tend to experience the highest mortality risk for stroke in the southeastern part of the country [4]. The higher risk of stroke in rural areas may be due, in part, to the higher proportion [8] of elderly people in the population and a higher prevalence of stroke risk factors. However, lack of timely access to emergency stroke care due to long travel times to stroke centers may also play a role [8]. Regional differences of stroke risk factors such as diabetes, high blood pressure, cigarette smoking, obesity and differences in socioeconomic factors may also explain some of the geographic differences in stroke mortality [7]. Additionally, differences in socio-demographic risk factors (e.g. socioeconomic status and race, etc) in addition to healthy behavior/lifestyle factors may play a role. A previous study noted that differences in educational attainment and income accounted for 32% of excess risk of stroke in the stroke belt in addition to the associated risk factors, such as hypertension and obesity [10]. And that even after adjusting for differences in common demographic risk factors, socioeconomic measures, behaviors such as smoking and physical activity, and other chronic conditions, geographical disparities still existed, implying that the causes of geographic disparities cannot be explained by differences in demographic and behavioral factors alone [17]. Moreover, studies performed in other states have shown that geographical disparities still existed even after adjusting for risk factors such as diabetes, hypertension, race, socioeconomic status [20, 21]. However, it is unclear if this holds true for Florida. Therefore, our subsequent studies will focus on these investigations. It is worth mentioning that the current study is the first of a series of studies that will investigate different aspect of stroke disparities in Florida to inform intervention efforts. Thus, the natural next step after this study will be to investigate the factors that help explain the disparities observed in the current study.

Statistically significant clusters/hotspots of stroke mortality were frequently observed in the northern region of Florida, which is consistent with findings from a previous study and therefore may provide evidence that north Florida could be a part of the United States stroke belt [21, 22]. These findings are consistent with other studies that have reported a higher burden of stroke in the southern region of the United States [23, 24] that includes northern Florida. Geographical disparities in stroke mortality have also been reported by Haverson et al [20] who investigated these disparities in Appalachian counties compared to other counties in the United States and reported that the Appalachian region exhibited higher rates of stroke mortality for all race/ethnic, gender, and age groups. They also reported that even within Appalachian counties, "economically distressed counties", as defined by the Appalachian Regional Commission, tended to have higher rates of stroke mortality than the rest of Appalachian counties.

A separate study of stroke in the US found that in white men and women aged 45–74 years, the risk of stroke was significantly higher in the Southeast than the Northeast or the West of the country [25]. The authors also reported that the observed excess risk could not be explained by regional differences in multiple stroke risk factors. However, in white women, some of the excess risk could be explained by the regional differences in risk factors measured by the study. It is interesting to note that the study did not find a statistically significant regional difference in blacks although higher stroke risk was observed among blacks living in nonmetropolitan areas. The study concluded that higher stroke incidence rates in the Southeast contribute to the higher stroke mortality rates in that US region [25].

Based on the findings from our study, there were significant low risk stroke mortality clusters in south Florida and this pattern was consistent throughout the study period. These lower risks are probably due to better access to care; an issue that will be investigated in future studies to be performed by authors of the current study. In the southern region of Florida, there are a large number of joint commission certified stroke centers (JCPSC). A study of Joint commission certified stroke centers reported that these centers tend to have lower stroke mortality rates than non-certified stroke centers [26]. The joint commission awards certification to primary stroke centers that comply with the national standards of care for stroke patients. Compliance with the national standards implies that the hospitals adhere to the expected clinical practice guidelines and performance measurement and improvement activities [26]. These facilities report on performance measures quarterly and an onsite visit is conducted every 2 years by the joint commission to ensure quality stroke care. Hospitals that achieve JCPSC certification are those that have met the critical elements to providing care that will improve stroke outcomes [27]. Thus, counties with consistently high mortality risk clusters tended to be in the north and may have poor access to certified stroke centers, since there are fewer of these centers in the north compared to the south. Similar to findings from this study, other studies in the US have also reported identifying significant geographic high rate clusters of stroke hospitalizations. For instance, a study by Schieb et al [28] investigated geographical disparities, over time, in stroke hospitalization rates in the United States and identified clusters of counties with high- and low-stroke hospitalization rates. They found that approximately 75% of counties studied retained their high rate cluster status from 1995–1996 to 2005–2006. The study also reported that 243 of the counties transitioned to high-rate clusters during the two time periods while 148 counties transitioned out of high-rate clusters. The others reported persistent high-rate clusters in the Southeast and persistent low-rate clusters in New England and the West of the country. Unlike the persistent high-rate clusters, persistently low-rate counties were also reported to have the most favorable socioeconomic and healthcare profiles. As mentioned earlier, this will be the focus of the next set of studies investigating geographical epidemiology of stroke in Florida.

A limitation of this study is that Florida is a premier tourist destination implying that the population changes based on the flow of tourists to and from the state. Moreover, these changes in population at-risk and residential population likely vary by region based on regional differences in tourist attractions. Given that central and south Florida tend to be the tourist destinations with northern Florida being less of a destination, it is quite possible that the true disparities might be larger than have been reported here. The problem of under-estimation of the true disparities may even be exacerbated by the fact that, in addition to tourists, Florida also serves as a seasonal home to “snowbirds” a population of elderly individuals that migrate from northern US states and Canada during the colder seasons in the north to seek warmer climates in the south. Thus the spike of stroke hospitalizations and deaths during the winter months may be due, in part, to the increased elderly population during these time periods. Thus, careful public health planning must be done to target healthcare systems in these regions.

The decreasing temporal trend observed in stroke mortality could be due to public health interventions such as improved access to certified stroke centers since the number of centers have increased over time. Similar to mortality risks overall stroke hospitalization risks in the US have also decreased and may be attributed to advances in acute stroke care [29]. A study by Towfighi et al [30] reported a decline in stroke mortality rates from 1997 to 2006 among individuals aged 35 to 64 years and explained that this was partly due to better survival among women compared with men aged 35 to 54 years. The seasonal patterns observed with high rates of stroke hospitalizations in the winter and early spring is consistent with findings from other studies [31, 32]. Higher stroke mortality risks in the winter may be attributed, in part, to physiological stresses that result from cold temperatures such as sympathetic activation and hypercoagulability resulting in increased stroke incidence [33].

## Conclusions

The current study demonstrates that there are dramatic disparities in stroke outcomes in Florida. The findings from this study help to identify high risk regions for future more detailed studies to identify specific risk factors that may be associated with the observed hotspots and to guide disease control efforts. We hypothesize that different risk factors may play significantly different roles in causing the observed patterns in different geographical locations. A better understanding of the factors associated with geographic disparities in the risk of stroke would help guide evidence-based strategies for disease control and prevention.

Traditionally, most past studies conducted by public health departments to investigate geographic disparities of diseases such as stroke have stopped at mapping the spatial distribution of disease risks/rates and not investigated if the observed disease risks/rates represent statistically significant clusters or not. Therefore, studies such as this one, that investigate significant clusters of disease burden such as stroke should be an integral part of the public health epidemiologist’s tool box to investigate community disease burden and identify high risk communities so as to better target limited resources to areas most at need with the goal of reducing/eliminating disparities. Finally, such analyses can be used to evaluate whether or not government-led interventions efforts reduce spatial disparities.
